# Thrombosis of the Vein of Trolard: An Atypical Presentation of Protein C Deficiency

**DOI:** 10.7759/cureus.50943

**Published:** 2023-12-22

**Authors:** Zeeshan Waqas, Saima Rahman, Sajid Khan, Arooba Khan, Yasir Ali, Iqbal Haider

**Affiliations:** 1 Internal Medicine, Khyber Teaching Hospital, Peshawar, PAK; 2 Radiology, Hayatabad Medical Complex, Peshawar, PAK; 3 Internal Medicine, Hayatabad Medical Complex, Peshawar, PAK

**Keywords:** restricted diffusion on dwi, blooming on gre sequence, thrombosis, vein of trolard, protein c deficiency, cerebral venous thrombosis cvt

## Abstract

Cerebral venous thrombosis refers to complete or partial occlusion of the cerebral sinus/es or the feeding cortical veins, resulting in secondary effects of vascular congestion and focal or generalized neurological deficits. One of the important causes of venous thromboembolism is inherited thrombophilia. Our case is of a 34-year-old male with no previous comorbidity who presented to the emergency department with complaints of sudden onset left-sided weakness, seizures, and loss of consciousness for one day. Thrombosis of the vein of Trolard was diagnosed based on magnetic resonance venography (MRV) film. His MRI with MRV revealed an attenuated caliber of the vein of Trolard along with abnormal signal intensity in the right fronto-parietal region and the right falcine location. He was managed with intravenous medication, including levetiracetam and topiramate. Once the diagnosis was established, he was commenced on subcutaneous Enoxaparin. Consequently, his GCS improved from 6/15 to 15/15 within the first 24 hours, and he could move his limbs on the day of discharge without any significant disability.

## Introduction

Cerebral venous thrombosis (CVT) is a life-threatening disease with significantly associated morbidity and mortality [1]. It has an incidence of about 5-8 cases per million [2]. The female-to-male ratio of incidence is 3:1, with the disease affecting individuals predominantly in the 25-35 age group [3]. The number of cases diagnosed has increased considerably with the advent of modern-day advanced imaging modalities like MR, magnetic resonance venography (MRV), and CT. Historically, females are more susceptible to developing this condition owing to hyper-coagulable states, including pregnancy, childbearing age, and the use of oral contraceptive pills (OCPs) [4].

## Case presentation

A 34-year-old male patient with an active lifestyle and no previous comorbidity was brought into the emergency department with complaints of left-sided weakness followed by complete loss of consciousness and seizures for the past one day. The seizures were generalized tonic-clonic seizures (GTCS) with urinary incontinence and tongue biting. He had no history of similar episodes, and his family history was unremarkable for cardiovascular diseases. On examination, he was vitally stable with blood pressure (BP) of 130/70mmHg and oxygen saturation (SpO_2_) of 93% on ambient air-the pulse of 113 BPM, which was regular, having normal volume and respiratory rate of 15 per minute. Additionally, a low Glasgow Coma Scale (GCS) of 6/15, complete uncrossed left-sided hemiplegia, and power of 0/5 in the left upper and left lower limbs were noted.

Furthermore, he had a positive Babinski’s sign on the left side. He was tachycardiac with a left pupil size of 3mm, which was sluggish in response to light. The rest of the systemic examination was unremarkable. Upon investigations (Table 1), he had a normal ECG and a normal echocardiogram with an ejection fraction (EF) of 60% with preserved left ventricular systolic function. Also, his Carotid Doppler ultrasound revealed normal findings.

**Table 1:** His laboratory investigations are tabulated above ALT: Alanine aminotransferase; AST: Aspartate aminotransferase; ALP: Alkaline phosphatase; INR: International normalized ratio; CRP: C-reactive protein

Investigations	Patients Values	Reference Ranges
Hemoglobin	9g/dL	12-16g/dl
ALT	39 U/L	10-50 U/L
AST	40 U/L	10-50 U/L
ALP	80 U/L	35-104U/L
Urea	35mg/dl	18-45mg/dl
Creatinine	0.8mg/dl	0.4-1.0mg/dl
Serum Sodium	137mmol/l	135-150 mmol/l
Serum Potassium	4.3mmol/l	3.5-5.1mmol/l
Serum Chloride	100mmol/l	96-112mmol/l
d-Dimers	2349mcg/l	Less than 0.5
INR	1	1
CRP	2.68mg/dl	Less than 0.5

**Figure 1:**
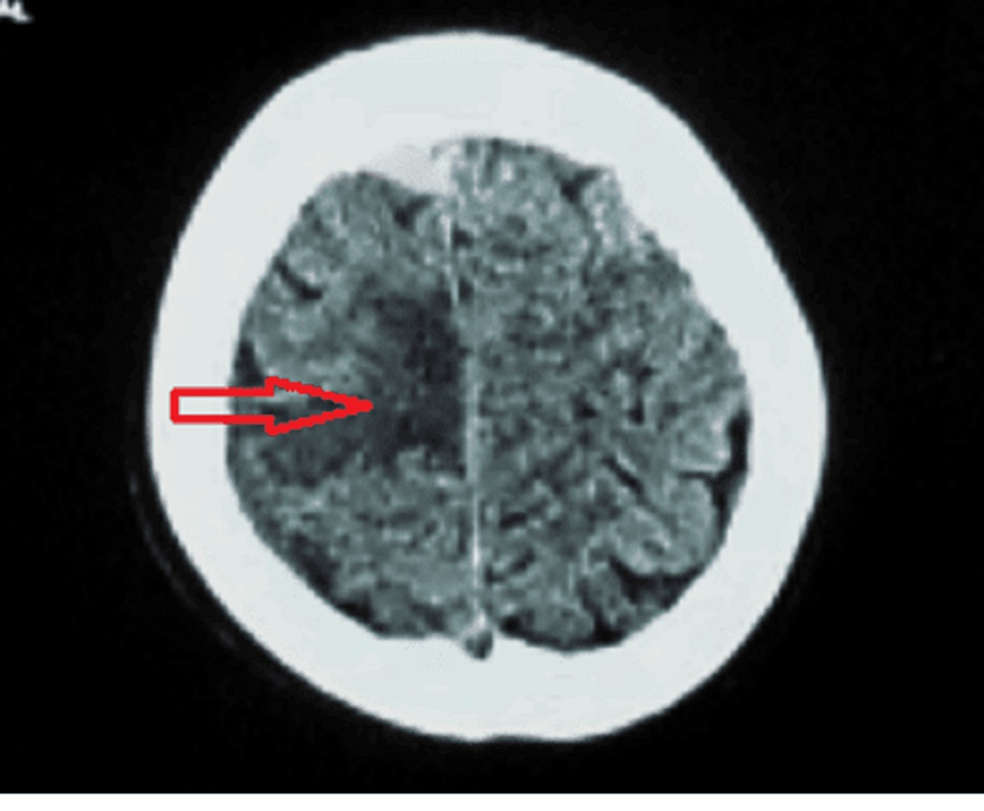
Non-Contrast CT-Scan Non-contrast CT brain showing hypodensity in the frontoparietal area

Upon initial resuscitation in the emergency department, he was advised to undergo a non-contrast CT, which revealed no bleeding. However, there was a hypodense area in the frontoparietal region suggestive of ischemia or a mass (Figure 1). Also, there was evidence of brain edema and intracranial hypertension in the CT scan film.

Consequently, he was started on IV 0.9% normal saline along with mannitol to relieve intracranial hypertension, which was revealed on his non-contrast CT scan. Levetiracetam was given to the patient to relieve his convulsions. The patient was consistently monitored with repeat GCS and National Institutes of Health Stroke Scale (NHISS) scores and vital monitoring. In light of no improvement in GCS, a repeat non-contrast CT brain was done, which revealed the same findings. His ECG and echocardiogram were normal, and he had no evidence of deep vein thrombosis (DVT) or pulmonary embolism (PE). The patient was admitted to the medical ward pending a blood work review. 

**Figure 2:**
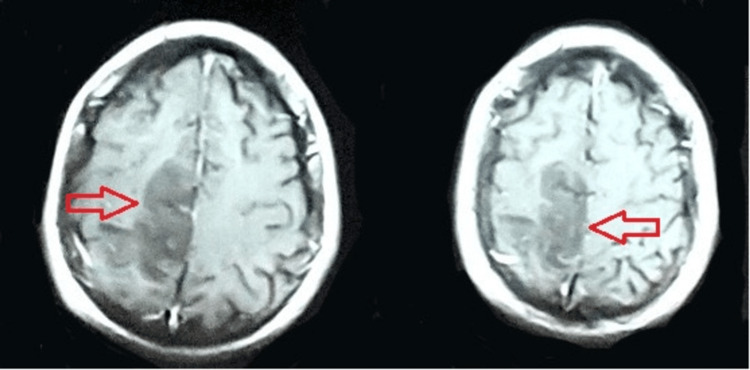
T1W MRI brain T1W MRI shows a hypo-intense lesion in the frontoparietal region. T1WI: T1 weighted image

**Figure 3:**
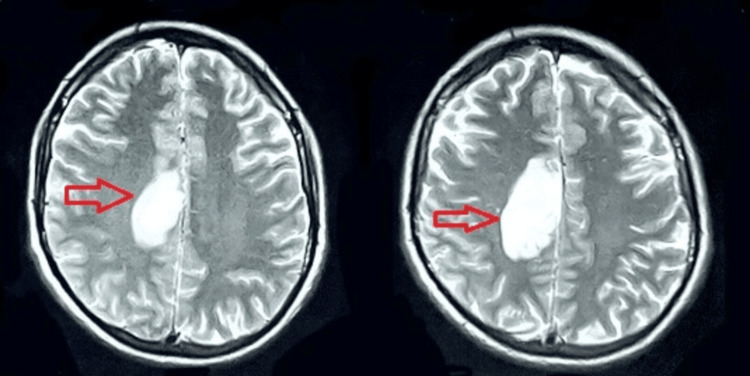
T2W MRI Brain Hyper-intense lesion in the frontoparietal region. T2WI: T2 weighted image

**Figure 4:**
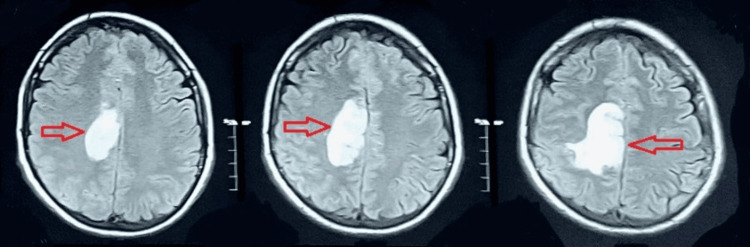
FLAIR MRI Bbrain A hyperintense lesion in the parietal region on FLAIR MRI. FLAIR: Fluid-attenuated inversion recovery

**Figure 5:**
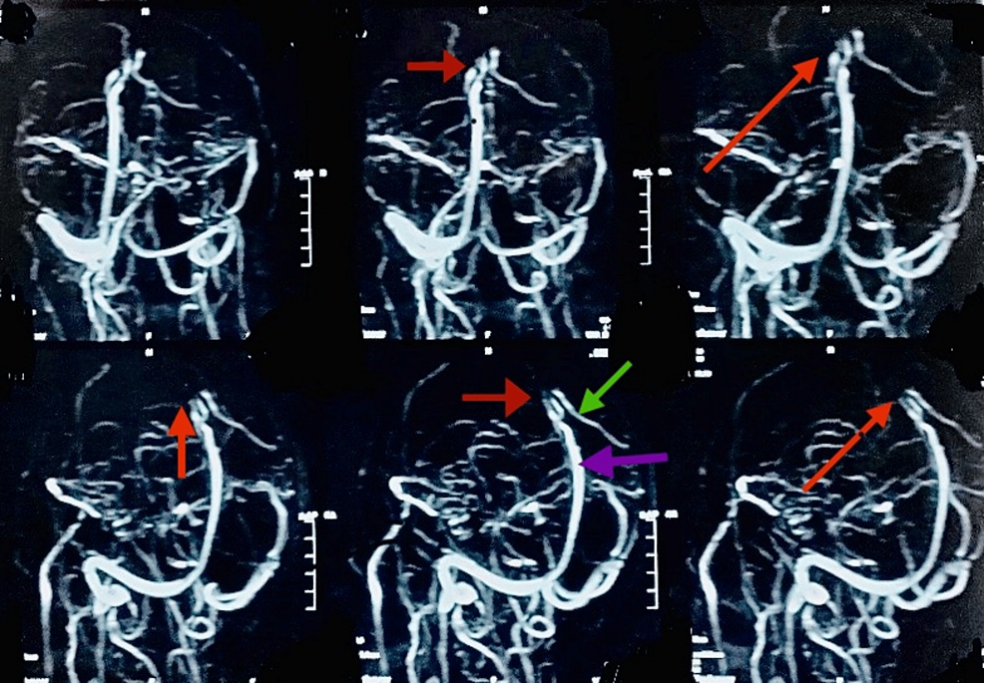
MRV Brain revealed attenuated caliber of the vein of Trolard with an abrupt cut-off at the site of confluence into superior sagittal sinus suggestive of thrombosis. The purple arrow indicates the Superior sagittal sinus, the green arrow shows a normal vein of Trolard, and the red arrow points to the affected (absent) vein of Trolard. MRV: Magnetic resonance venography

In the medical unit, a neurological consult was arranged for him, and upon assessment by a consultant neurologist, he was advised to undergo an MRI with MRV. His MRI revealed hypo-intensity on the T1 weighted image (T1WI), hyper-intensity lesion on the T2 weighted image (T2WI) and fluid-attenuated inversion recovery (FLAIR) images(Figures 2-4), restricted diffusion on diffusion-weighted imaging (DWI), and blooming on multiecho gradient recalled echo (GRE) sequence In the right frontoparietal region suggestive of venous-hemorrhagic infarct. Furthermore, his MRV revealed an attenuated caliber of the vein of Trolard with an abrupt cut-off at the site of confluence into the superior sagittal sinus, suggestive of thrombosis (Figure 5).

Consequently, he was commenced on anticoagulation with Enoxaparin. Fortunately, within 24 hours of therapy, his GCS improved to 15/15 with a power of 3/5 in the left upper limb. The left lower limb still had a power of 0/5. Headache and vomiting persisted, so a repeat CT scan was ordered that revealed an extension of edema. As a result, he was commenced on topiramate, which relieved the cerebral edema.

To find the underlying cause of thrombosis, an inherited thrombophilia screen was requested 4-weeks later. The reports revealed normal titers of protein-S, absent factor-V Leiden, and prothrombin gene mutation but decreased titers of protein-C measuring 40IU/L with a reference range of 65-115IU/L.

## Discussion

Protein-C is a liver-produced glycoprotein whose synthesis relies on vitamin K [5]. It acts as an anti-coagulant by inactivating coagulation factors Va and VIIIa, which are required for factor X activation. The gene for protein-C is located on the long arm of chromosome 2, and nearly two hundred mutations have been described in this [6]. Protein-C deficiency affects 1 in 500 persons [7]. In patients presenting with venous thromboembolism (VTE), approximately 3 to 5% may have protein-C deficiency [8,9].

The most common sites of thrombosis due to protein-C deficiency include deep veins of the lower extremities, mesenteric vein, and cerebral venous sinuses [10]. CVT normally affects the transverse sinus in 86%, superior sagittal sinus in 62%, straight sinus in 18%, cortical veins in 17%, jugular veins in 12%, vein of Galen and internal brain veins in 11% of the cases [11].

Isolated unilateral thrombosis of the vein of Trolard is a rare finding in cases of CVT [12]. It is an important cortical vein as it drains the eloquent cortex. The modality of choice in CVT is an MRI with MRV that has led to early diagnosis and prompt intervention, further reducing morbidity and mortality worldwide.

Anticoagulation is the mainstay of treatment according to American Heart Association/American Stroke Association (AHA/ASA) guidelines. Thrombolysis/ Thrombectomy is indicated according to AHA/ASA guidelines if the patient fails to improve or clinically deteriorates despite anticoagulation for a minimum of 24 hours.

## Conclusions

Isolated unilateral involvement of the vein of Trolard is a primary presentation of Protein-C deficiency with variable symptoms, including hemiplegia, seizures, and raised intracranial pressure are an important dilemma that requires early diagnosis and prompt intervention as it can have significant debilitating consequences. Early anticoagulation with Enoxaparin in the initial 24 hours of presentation showed a dramatic reversal of the symptoms and limited the risk of complications.
